# Convolutional architectures for virtual screening

**DOI:** 10.1186/s12859-020-03645-9

**Published:** 2020-09-16

**Authors:** Isabella Mendolia, Salvatore Contino, Ugo Perricone, Edoardo Ardizzone, Roberto Pirrone

**Affiliations:** 1 Dipartimento di Ingegneria Universit’a degli Studi di Palermo, Viale delle Scienze, Edificio 6, 90128 Palermo, Italy; 2 Gruppo Drug Design, Fondazione Ri.MED, 90133 Palermo, Italy

**Keywords:** Deep learning, Drug design, Molecular fingerprints, Bioactivity prediction, Virtual screening

## Abstract

**Background:**

A Virtual Screening algorithm has to adapt to the different stages of this process. Early screening needs to ensure that all bioactive compounds are ranked in the first positions despite of the number of false positives, while a second screening round is aimed at increasing the prediction accuracy.

**Results:**

A novel CNN architecture is presented to this aim, which predicts bioactivity of candidate compounds on CDK1 using a combination of molecular fingerprints as their vector representation, and has been trained suitably to achieve good results as regards both enrichment factor and accuracy in different screening modes (98*.*55% accuracy in active-only selection, and 98*.*88% in high precision discrimination).

**Conclusion:**

The proposed architecture outperforms state-of-the-art ML approaches, and some interesting insights on molecular fingerprints are devised.

## Background

Virtual Screening (VS) is a routinely applied computational technique useful for drug design. However, some issues remain uncertain due to the complexity of the algorithms used behind the screening campaign, and this leads to generate models with different prediction reliability. Clinical candidate molecules selected by drug detection must have a profile responding to different criteria, that are based not only on the effect potency but also on the selectivity, safety as well as the so called *ADMET* properties (Absorption, Distribution, Metabolism, Excretion and Toxicity). Therefore, the design of the optimal compound is a multidimensional challenge involving different aspects of Chemistry and Biology, which can be faced using Machine Learning (ML).

One key aspect for ML approaches gaining success in property prediction, is the possibility to access and mining large data sets that contain heterogeneous information. Until recent years, the best performing ML techniques were “shallow” ones [1] that is Support Vector Machines (SVM) and decision trees, particularly ensemble methods like Random Forests (RF).

All these ML models should be iteratively refined with new experimental data to increase model reliability and predictive power. In the last few years, Deep Learning Techniques, and particularly Convolutional Neural Networks (CNN) gained more and more impact on drug design and VS due to the huge increase of the prediction accuracy in any stages of this process [2, 3]. Deep Neural Networks (DNN) have been used for predicting different properties such as biological activity, ADMET properties, and physicochemical parameters demonstrating reliable and robust prediction capabilities with high sensitivity when used on different targets [4, 5]. CNNs have been used also to predict several properties such as the kinetic energy of hydrocarbons as a function of electron density [6]. Several DNN architectures use Simplified Molecular Input Line Entry System (SMILES) as their input data [7–9]. SMILES is actually a simple chemical language whose rules allow building string descriptors that can represent both molecular structures and reactions.

One of the most frequently asked questions by computational chemists, is if it is better to have a model retrieving some false positives or to loose some actives as false negatives. Depending on the drug discovery phase, at the beginning of the drug discovery cascade, it could be useful to have some false positives, instead of losing some putative hits. In a more mature screening stage (e.g. hit expansion) it could be better, instead, to have more precise algorithm preventing the discovery of false positives. Based on these considerations, the best compromise is a virtual screening model that can adapt to the drug design campaign stage.

In this work we present a novel CNN VS of candidate compounds with respect to their biological activity on the Cyclin-Dependent Kinase 1 (CDK1) target. Vector representation of candidate compounds is achieved using their molecular fingerprints. The choice of this target is given by the previous experience of the research group in the CDK1 modulators and the fact that canonical VS approaches on CDKs do not respond properly to activity prediction because of high structural similarity between different kinases’ binding sites. The importance of the target is given by its validation as drug target. It is an archetypal kinase acting as central regulator that drives cells through G2 phase and mitosis. Its importance in tumorigenesis has been demonstrated by the evidence that, unlike other CDKs, loss of CDK1 in the liver confers complete resistance against tumor formation demonstrating its role in the cancer development [10]. The Kinase protein family presents a huge variety, and contains a very high number of proteins so it provides an amount of data that is well suited for ML approaches. In [11] Bayesian models were generated for building Quantitative Structure-Activity Relationship (QSAR) models on different kinases from a large, but sparsely populated data matrix of more than 100,000 compounds. Random Forest has been applied in another case study for predicting kinase activities on hundreds of kinases starting from publicly available data sets integrated with in-house data [12]. In several examples, Random Forest models showed a higher reliability in prediction when compared to other approaches, but they perform worse than Deep Neural Networks (DNN).

The work presents two qualifying points:

- molecular fingerprints are used as a suitable embedding for describing molecules;
- a unique neural architecture has been designed, which has been trained with different hyperparameters for achieving good performances in both early and mature screening.

The proposed approach is quite novel; up to our knowledge, there are a very few and recent examples using deep learning and fingerprint in a VS workflow [13]. An interesting approach is presented by Hirohara et al. who present a CNN that learns a suitable fingerprint from SMILES, and use such a feature to classify both active and inactive compounds [14]. As regards the proposed application, the literature reports very few recent approaches for Virtual Screening with respect to Cyclin-Dependent Kinase proteins that do not use molecular fingerprints as their descriptor [15, 16]. Finally, DNN-VS is a very recent network for VS applied to the Tyrosine Kinase using molecular descriptors [17]. Indeed, molecular fingerprints are the most natural choice for describing compounds as inputs for a neural network, due to their inherent structure of numerical vectors that encode all the substructures inside a molecule.

**Fig. 1:**
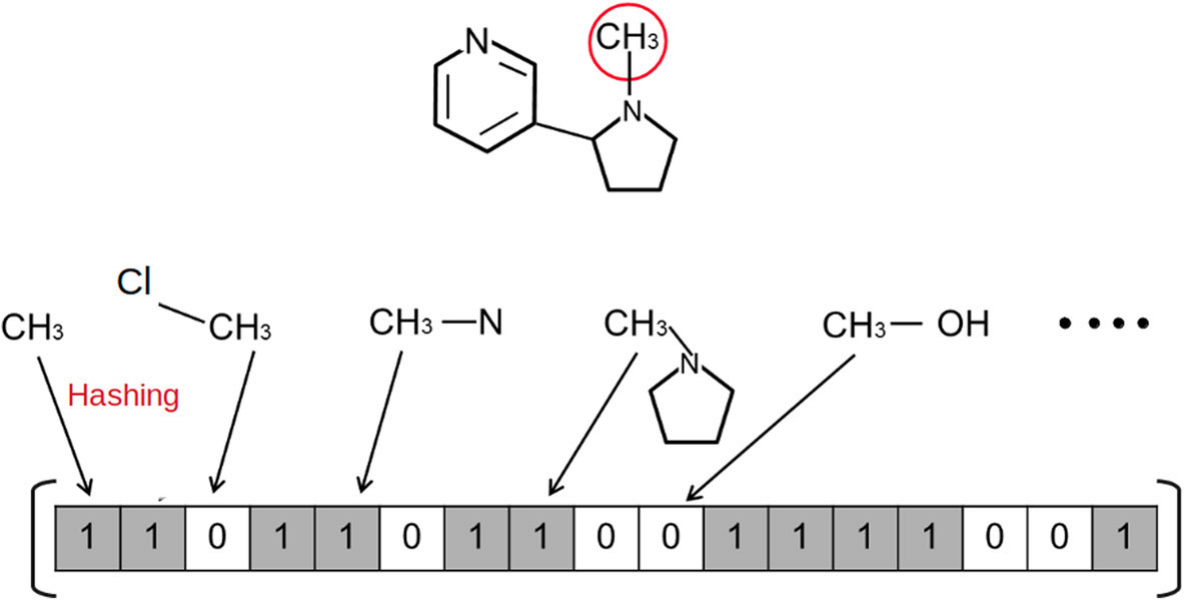
Fingerprint Generation. Simplefied Fingerprint generation, the hashing function sets just 1 bit per pattern

Molecular fingerprints are binary vectors, which are generated analyzing each atom along with its neighborhood till 6 or 7 bonds away. Such a neighborhood is searched for a set of predefined molecular substructures, the so called *patterns*, that is atom types, bond types, presence of rings, and so on. After having enumerated all the patterns in the molecule, each of them is used as a seed for a hashing function that outputs in general 4 to 5 index positions whose corresponding bits are set to 1 in the “pattern fingerprint”; such a fingerprint is bit-wise OR ed to the molecular one. Actually the hashing function can cause a bit collision so we are not guaranteed of the effective presence of a particular pattern unless at least one of its bits is unique. On the other hand, a molecular substructure is absent if all its bits are set to 0 in the fingerprint. A simplified fingerprint generation procedure is reported in Fig. 1.

Several kinds of fingerprints with different sizes are reported in the scientific literature to address different aspects of both the structure and the local properties of a molecule [18]. In this work, we dealt with seven of the most popular fingerprint types: *RDKit*, *Morgan*, *AtomPair*, *Torsion*, *Layered*, *FeatMorgan*, *ECFP4*. A substantial part of this work was devoted to address either the single fingerprint or the fingerprint combination that allows achieving the best accuracy in both a highly discriminative task (i.e. mature screening) and an active-only selection task (i.e. early screening).

A molecular fingerprint represents the corresponding molecule “as a whole” in a suitable vector form, that is it conveys information about the presence of a particular substructure, but not on its exact position or its repetition in different sites of the same molecule. Moreover, we aimed at performing a binary classification between active and inactive compounds, and biological activity is mostly related to the presence/absence of particular substructures which in turn are well suited to bind to the target protein. As a consequence, CNNs appeared to be the best architectural choice to classify molecular fingerprints. On the other hand, different fingerprint types address different aspects of the molecular structure through different search strategies. A single fingerprint might not make explicit the particular substructure that is responsible for binding to the target, and this is due to both its search strategy and the hashing mechanism. One of the key ideas of this work is that many fingerprints used together to describe the same candidate compound can make explicit the features responsible for bioactivity. Moreover, a neural model with adequate capacity can accommodate for the redundancy derived from having the same molecular pattern encoded in different fingerprints.

In view of the previous considerations, both 1D and 2D CNNs have been trained to test the performance of each single descriptor separately, along with all the combinations of multiple descriptors for the same compound. Moreover, also two complex architectures have been designed. The first one uses the best performing 1D CNNs in a direct voting scheme, and we called it *Voting*. The second architecture uses a 1D CNN for each fingerprint type; such networks were retrained purposely to act as inputs of a Multi Layer Perceptron (MLP) classifier. We called this last architecture *Tuned-MLP-Out*. Each network has been trained with different hyperparameter tuning to perform both discriminative and active-only classification. All the architectures are described in detail in the section entitled The proposed architectures, and the results are compared against traditional Machine Learning (ML) approaches.

## Results and discussion

We performed two kinds of experiments as regards the training procedure, and several measures were collected to devise performance in both tasks. The first training procedure (*training scheme 1*) makes use of a classic ML approach for training where the ratio between training, validation and test set is strongly biased towards the training set, while keeping the ratio between the two classes untouched in all of them. This is the correct choice if one wants to maximize the discriminative power of the network. On the other hand, the second procedure (*training scheme 2*) takes into account that the general population of a data set containing candidate compounds to be screened is strongly biased towards inactive candidates. As a consequence, we stressed the performance of the network using a balanced training with many active compounds, and testing with a 1 : 50 active/inactive ratio. Both the schemes make use of a 10-fold cross-validation in the training phase.

The balanced accuracy *bACC* = (*TP/P* + *TN/N*)*/*2 has been used for active/inactive discrimination, which is a binary classification task. The *bACC* value is the mean of sensitivity or *true positive rate* that is the ratio between the predicted positives *TP* and the labeled positives *P*, and specificity or *true negative rate* that is the ratio between the predicted negatives *TN* and the labeled negatives *N*. *bACC* measures the performance in labeling each sample in the proper class. Also the *Matthews correlation coefficient* (MCC) was used as a discrimination measure. MCC is a well known index used for binary classification, that returns a value in [−1;1]; for a 2 × 2 contingency table, that is a binary classifier’s confusion matrix, *MCC* is related to the chi-square statistic as $$ \left\Vert MCC\right\Vert =\sqrt{\raisebox{1ex}{${X}^2$}\!\left/ \!\raisebox{-1ex}{$n$}\right.} $$ where *n* is the number of observations. *MCC* thus measures the dependency of the predictions from the true (i.e. expected) labels. On the other hand, just sensitivity has been used in the active only selection task because we want to maximize correct prediction of active compounds in spite of accepting a relevant number of false positives.

Our models are compared with two state-of-the-art ML approaches for Virtual Screening that is Support Vector Machines (SVM), and Random Forests (RF) which form the baseline for our experiments. The parameters for both models where devised using a classical grid search. Particularly, a Radial Basis Function-SVM has been trained, and the best performing machines have *γ* = 1 for both training schemes, while the regularization parameter is *C* = 5 in the training scheme 1, while *C* = 1 and *γ* = 0*.*1 in the training scheme 2. SVM trained on *FeatMorgan* fingerprint performed the best in both the training schemes. The best performing RF used 100 estimators, and the *Gini* index for the training scheme 1 on the *FeatMorgan* fingerprint, while in training scheme 2 *Gini* index and 2 estimators.

The results of the best performing architecture for each task are reported in

Table 1 and Table 2. The two tables show clearly that the reduced number of samples in the training scheme 2 along with the inherent class unbalancing in the data set reduce the absolute performance of the network due to an increase of false negatives. Even if both *bACC* and sensitivity are acceptable, the *Loss* value doubles with respect to the training scheme 1. This reflects on all the global measures that are *AUC*, *F*_1_ score, and *MCC*. The winning architecture for both tasks is *TunedMLP-Out* because it takes into account all the fingerprint types, and manages the possible redundancies by training a shallow MLP classifier. Just one layer was always sufficient to achieve good classification, even if we tried different sizes for the hidden layers. Particularly, discrimination task was performed with 3 units, learning rate equal to 10^−3^, and Adam optimizer, while active-only selection was accomplished using 5 units, learning rate equal to 10^−4^, and Adamax optimizer. Active-only selection is achieved with a classifier with both higher capacity and lower learning rate than in the discrimination case. These values indicate a network that is more prone to overfitting than in the balanced case as it is less accurate. Also the 1D CNNs used by *Tuned-MLP-Out* have higer capacity than the best performing 1D CNNs alone.

**Table 1:** Results for the active/inactive discrimination task, and Training scheme 1

Architecture	Bal. accuracy	Sensitivity	Loss	AUC	F1-score	MCC
Tuned-MLP-Out	0.9880	0.9855	0.0405	0.9979	0.9510	0.9462
Voting	0.9768	0.9710	0.2093	0.9920	0.8965	0.9033
CNN 1D (F)	0.9687	0.9710	0.0688	0.9904	0.8979	0.8813
CNN 2D (R-M-F)	0.9679	0.9565	0.0770	0.9912	0.8918	0.8817
Random Forest (F)	0.9510	0.8985	0.6405	0.9837	0.6065	0.8962
SVM (F)	0.9421	0.8985	0.7883	0.9868	0.8857	0.8731

Fingerprint types: *(R)DKIT*,*(M) organ*, *(F) eatMorgan*, *(L)ayered*

**Table 2:** Results for the active/inactive discrimination task, and Training scheme 2

Architecture	Bal. Accuracy	Sensitivity	Loss	AUC	F1-score	MCC
Tuned-MLP-Out	0.9644	0.9625	0.0983	0.9875	0.5519	0.5989
Voting	0.9639	0.9500	0.1523	0.9889	0.6379	0.6694
CNN 1D (F)	0.9579	0.9625	0.1398	0.9854	0.4709	0.5336
CNN 2D (T-L-E)	0.9525	0.9375	0.1054	0.9841	0.5192	0.5920
Random Forest (F)	0.8789	0.7750	0.6221	0.9541	0.6528	0.6540
SVM (F)	0.9208	0.8625	0.6221	0.9682	0.6699	0.6524

Fingerprint types: *(F) eatMorgan*, *(T) orsion*, *(L) ayered*, *(E)CFP4*

The winning *Voting* architecture is the same for both tasks because it uses always the best 1D CNN for each fingerprint type. This network exhibits always the highest *AUC* value, which means that it tends to have a good balanced performance in every case. In fact, *Voting* has the lowest sensitivity when used for active-only selection, and it falls below the baseline, but it is one of the best ranked networks in terms of the *bACC* value.

A common performance measure used in VS procedures is the so called *Enrichment factor* (*EF*) which measures the number of predicted true actives, in decreasing probability order, in a fixed percentage of the test set. Typical percentage values are 5% and 10% but also smaller values are used. EF is crucial in VS procedures due to the huge number of candidates to be evaluated, so the drug designers require that a good VS procedure assigns the highest probability values to the very first candidates in the data set, in order to discard the remaining ones without further test. We compared our best performing architecture versus both SVM and RF also as regards the *EF* value. Results are reported in Table 3, and Table 4 respectively for each training scheme. In particular, in training scheme 1 we were able to compute *EF* from 1% to 10% because both training and test set were equally balanced. Just *EF*1%, and *EF*2% were computed in the training scheme 2 because only 80 out of 3270 molecules were truly active that is a percentage of 2*.*4%. As a consequence, computing both *EF*5% and *EF*10% would have resulted in a artificial performance decrease by definition. Results are satisfactory. Our *Tuned-MLP-Out* network ranks at 100% in *EF*1% and *EF*2%, just like RF and SVM. It is worth noticing that drug designers are more interested computing *EF* for low percentages that implies screening very few candidates. For high percentages, the probability of a false positive prediction increases. Even if, our architecture misses just one active compound with respect to both SVM and RF in the case of *EF*5% both the shallow models exhibit a low *EF*10% value due to their reduced accuracy on the whole data set.

**Table 3:** Enrichment factor computed on the test set 1 (70 active molecules out of 701 compounds)

Architecture	EF 1%	EF 2%	EF 5%	EF 10%
Tuned-MLP-Out	7(100%)	14(100%)	34(97.14%)	65(94.20%)
Voting	7(100%)	14(100%)	34(97.14%)	61(88.40%)
CNN 1D (M)	7(100%)	13(92.86%)	33(94.29%)	62(85.89%)
CNN 2D (R-M-F)	7(100%)	12(85.71%)	32(91.43%)	61(88.40%)
RF(F)	7(100%)	14(100%)	35(100%)	63(91.30%)
SVM(F)	7(100%)	14(100%)	35(100%)	61(88.40%)

Fingerprint types: *(R)DKIT*,*(M) organ*, *(F)eatMorgan*,

**Table 4:** Enrichment factor computed on the test set 2 (80 active molecules out of 3720 compounds)

Architecture (Training 2)	EF 1%	EF 2
Tuned-MLP-Out	37(100%)	65(87.84%)
Voting	32(86.5%)	57(66.9%)
CNN 1D (F)	31(83.8%)	52(70.3%)
CNN 2D (T-L-E)	31(83.8%	52(70.3%)
RF(F)	37(100%)	62(83.8%)
SVM(F)	32(86.5%)	55(74.3%)

Fingerprint types: *(F) eatMorgan*, *(L) ayered*, *(T) orsion*, *(E)CFP4*

Experiments gave us some interesting insights on the use of different fingerprint types. The best performing 2D CNN is the one using the combination of *RDKit*, *Morgan*, and *FeatMorgan* fingerprints. Such a network has a good mix of accuracy, sensitivity, and a high *AUC* together with a very low Loss value. As already pointed out in the previous section, the network has a good general behaviour but it can not be pushed towards extreme performance in neither task we addressed in this work. Finally, 1D CNNs differ only in the fingerprint type used in the training phase. The *Layered* network showed the best *bACC*, while the *Morgan* one has the highest sensitivity. The reason for this difference in predictive ability lies in the different way of interpreting the molecular structure. The *Layered* fingerprint, using different layers of structural analysis, seems to outperform in discriminating between active and inactive candidate compounds. The *Morgan/FeatMorgan* fingerprints represent a circular approach which uses either connectivity or feature invariants, and it has been outclassed by modern ECFP fingerprints as they are more accurate. Nevertheless, both 1D and 2D CNNs have the best performance when such descriptors are used to represent the candidate compounds. It is worth noting, in this respect, that in this work, we have tested the ability to recognize between active and inactive molecules, based on their *IC*_50_ value, and such a task requires a more discriminative power than other works in the literature in which fingerprints are compared on the basis of the distinction between actives and decoys.

Our approach has to be regarded as typical ligand-based one, while decoys are generated to validate docking-based algorithms. On the other hand, decoys are synthetic molecules whose mere structure could make them active on the target, and their use to train an active/inactive classifier could result in a poor discriminative power. Even if it is well known in the literature that a 1 : 10 active/inactive ratio is a common value for *in silico* screening, we performed some stress tests on our best architecture that is *Tuned-MLP-Out* with training scheme 1. We aimed at devising its performance in a typical *in vivo* screening, where several problems can occur in essays, thus reducing the active/inactive ratio even to 1 : 100. We resampled our data set to obtain three different data sets with varying active/inactive ratio: 1 : 20, 1 : 50 and 1 : 100 respectively. The *Tuned-MLP-Out* network was trained using the scheme 1 on all of them. The results of active/inactive discrimination with these different proportions are reported in Table 5 while EF values from 1% to 5% are reported in Table 6. As expected, all the *bACC*, *Sensitivity*, and *EF* values decreased with respect to the results reported in Table 1. This is due to the extreme unbalancing between classes that is a hard issue for whatever learning algorithm. Nonetheless, the results are still positive, and this can be observed in Table 6. Apart from the *EF*1% for the 2% proportion data set that attains a 87*.*5% value, almost all the *EF* values drop to values that are close to 50%, but all the hits in whathever experiment ranked as the very first molecules in terms of the output probabilities of the model so they still remain the first choice for the drug designer. Also in this case we did not compute *EF* values for test percentages greater than the true active/inactive proportion to avoid the computation of false low values due to the absence of active molecules.

**Table 5:** Performance of the *Tuned-MLP-Out* network on three data sets with 1%, 2%, and 5% active/inactive proportion respectively

Active/inactive proportion	Bal.Accuracy	Sensitivity	Loss	AUC	F1-score	MCC
1%	0.7475	0.5000	0.5116	0.9700	0.5333	0.5289
2%	0.9671	0.9375	0.5114	0.9415	0.9009	0.8226
5%	0.9382	0.8780	0.0565	0.9991	0.9230	0.9196

**Table 6:** Enrichment factor computed on the test set with 1%, 2%, and 5% active/inactive proportion respectively

Active/inactive proportion	EF 1%	EF 2%	EF 5
1% (8 active molecules)	4(50%)	–	–
2% (16 active molecules)	7(87.5%)	7(43.5%)	–
5% (41 active molecules)	4(50%)	8(50%)	20(48.7%)

## Conclusions

A novel CNN architecture has been presented in this work, that is trained on the molecular fingerprints to predict biological activity of candidate medical compounds versus the CDK1 protein target, using their *IC*_50_ value. The paper contains two main novelties. The first one is using molecular fingerprints as the embedding for a VS deep neural architecture both alone and combined together as bidimensional binary matrices. The second novelty is designing several architectures purposely for achieving good performances in both early screening, when almost all active candidates have to be selected in spite of having many false positives, and a mature stage, when we want a precise discrimination between active and inactive molecules. Four architectures were developed: 1D and 2D CNN classifiers for both single fingerprints and suitable combinations, a voting scheme based on the 1D CNN classifiers, and a an architecture that classifies the output of different 1D CNNs using a MLP trained purposely. The results outperform the state of the art ML approaches. Our experiments gave us very interesting hints on the role of each different fingerprint type in Virtual Screening based on deep neural architecture. Future work will be oriented towards three main goals. At first, we want to deepen the study of the influence of each fingerprint on the screening accuracy, while trying to devise which molecular substructures (i.e. fingerprint patterns) are responsible for bioactivity. Next, we are designing a wide classifier for multiple Kinase families to cope with all the targets involved in the cell cycle. Finally, we want to design a suitable descriptor aimed at conveying detailed information on the position of relevant molecular substructure, while retaining the compactness of molecular fingerprints.

## Methods

### Data representation

The data used in our experiments were extracted from the well known CheMBL molecular database [19]. Biological activity of the tested compounds was measured using the *half maximal inhibitory concentration* parameter (*IC*_50_) that is the amount of substance which is needed to inhibit the target protein by one half. The literature does not report a precise threshold to be used for labeling a compound as *active* or *inactive*. A good rule of thumb is that *IC*_50_ *<* 1*.*0 *μM* implies good bioactivity, while *IC*_50_ *>* 10*.*0 *μM* indicates definitely no bioactivity. Our task is a binary classification so we needed a crisp threshold to divide our data in two classes. As a consequence, we followed a typical ML approach in this respect, that is we devised the threshold from the data using K-Means clustering. We didn’t have any knowledge in advance about the distribution of the *IC*_50_ values in our data. At first, the so called *elbow method* was applied to assess the correct number of clusters, running the K-Means algorithm for a variable number of tentative clusters, and plotting the *Within Cluster Sum of Squares* value (*WCSS*) against the cluster number. The heuristic rule used in this case says that one has to select the cluster number “where the plot has an elbow”. Our analysis resulted in choosing two clusters, as we were expecting. Then we ran the K-Means algorithm with two clusters, and we obtained a threshold value for *IC*_50_ = 7*.*414 *μM*, so a molecule was labeled *active* when *IC*_50_ ≤ 7*.*414 *μM*. It is worth noticing that this value was used merely for splitting the data in two classes. There is no chemical relevance in this threshold. Actually, it is the value of the two class centroids’ average. The KMeans algorithm reported also the following results about the shape of the clusters, that are coherent with the literature:

- Active molecules: centroid at *IC*_50_ = 0*.*91762 μM, upper bound at *IC*_50_ = 0*.*971 *μM*
- Inactive molecules: centroid at *IC*_50_ = 13*.*91221 *μM*, lower bound at *IC*_50_ = 13*.*338 *μM*

We used the KNIME data analysis platform [20], to implement a preprocessing workflow for both the training and the test set. Activity data for 1830 compounds on the CDK1 target were taken from the *CHEMBL308* ID were CDK1 is considered as a single protein, and the *CHEMBL1907602* ID were it is considered as a protein complex. At first, incomplete data were deleted; the resulting data set was then made by 1720 samples. The data set was expanded using some compounds, which are active on some kinases with very different structure from CDK1. Also these molecules were extracted from CheMBL. In particular, we selected 2422 active compounds on TRKA (Tropomyosin receptor kinase A, CHEMBL2815), 50 active compounds on RIPK1 (Receptor-Interacting Protein 1, CHEMBL5464), 2825 active compounds on AKT1 (AKT Serine/Threonine Kinase 1, CHEMBL4282), and 199 active compounds on LIMk1 (LIM Domain Kinase 1, CHEMBL5932). Duplicates have been removed from the original 5496 records returned by the queries thus obtaining 5452 inactive compounds on CDK1.

Two different schemes have been used for training even if 10-fold cross-validation has been used in both procedures. In the scheme 1, we adopted a classic strategy with an approximate 80%:10%:10% split for training, validation, and test set respectively with a 1 : 10 active/inactive ratio. Validation set has been used for hyperparameters grid search while Test set has been used to evaluate the overall performance. In the scheme 2, the same data set as above has been divided in two almost equal parts (48% training, and 52% test set). Moreover the training data were split in training and validation set with a 90%:10% ratio. In the training data 720 compounds out of 3440 samples were active molecules, while just 80 active molecules out of 3720 compounds were present in the test set.

Finally we turned each fingerprint’s 0 value in -1 to cope with the inherent sparsity of such a vector representation. In this way we maintained the binary information conveyed by each fingerprint, while avoiding unwanted bias of the output of the convolutional units when they receive an almost zero input.

### The proposed architectures

The 1D CNNs were trained on single fingerprints; seven networks were trained, one for each tested fingerprint type. We selected only 1024 bit fingerprints as a good trade-off between compactness and expressivity. Low size fingerprints are too small to allow the network learning their features properly, while 2048 or 4096 bit fingerprints require models with very high capacity whose training is difficult. In the 2D CNNs each compound was represented by a combination of different fingerprints arranged as a bi-dimensional {1; −1} matrix. The 2D CNNs have been trained on all possible combinations, ranging from fingerprint couples to a unique 7 × 1024 matrix enclosing all the fingerprints in a single descriptor. The intuition behind this architecture is that a fingerprint ensemble represents in a single tensor all the structural properties of a compound that concur to its bioactivity on the target. On the other hand, a fingerprint combination can attain a high redundancy because the same pattern is encoded in several rows of the resulting matrix so we are not guaranteed that the more fingerprints are present in the 2D descriptor the more accuracy we will obtain after training the network.

Both 1D and 2D networks consist of 4 convolutional layers with 128*/*64*/*32*/*16 filters per layer, and ReLU activation, each followed by a 2x2 Max Pooling, while they differ only in the convolutional kernel dimensions. Such networks have 512*/*256*/*128*/*64 filters respectively for each convolutional layer, while the number of filters per layer in the 1D CNNs used for direct classification are 128*/*64*/*32*/*16.

**Fig. 2:**
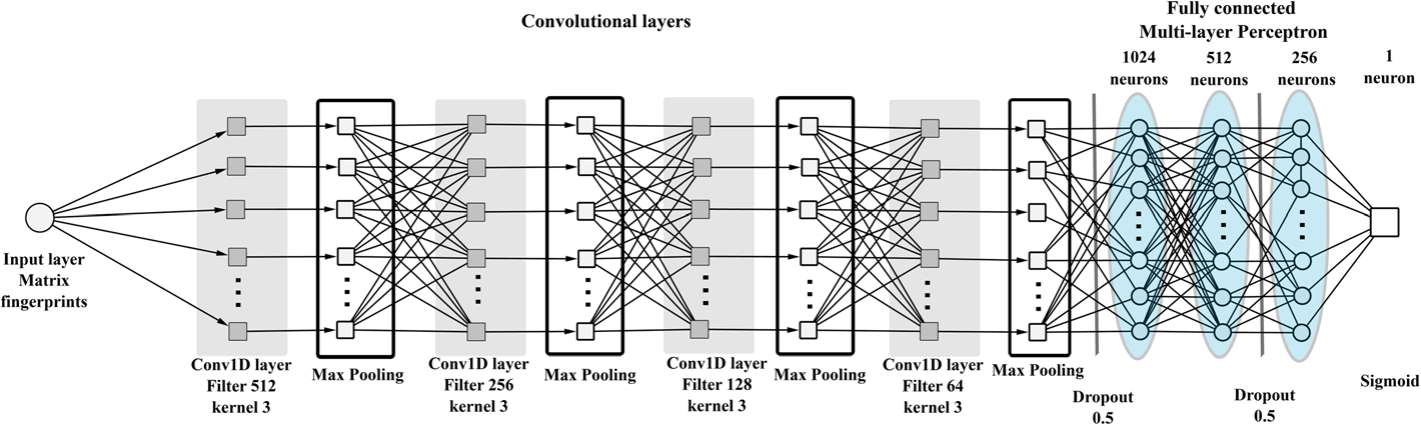
1D CNN. One-dimensional convolution architecture

**Fig. 3:**
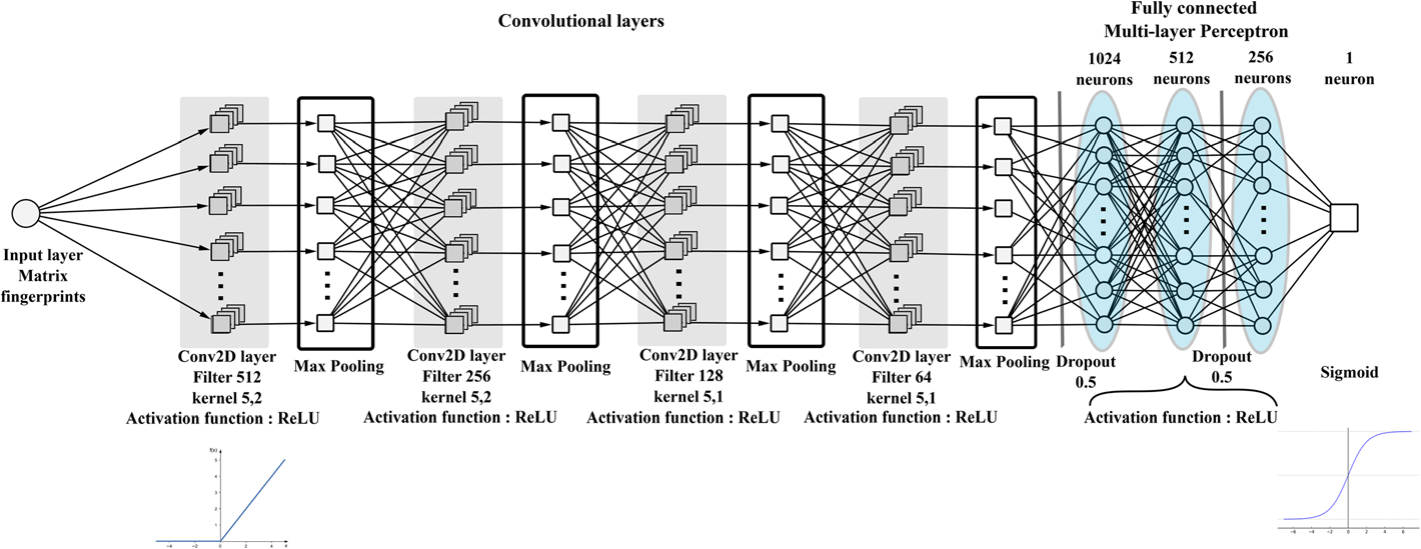
2D CNN.Bi-dimensional convolution architecture

Classification is achieved through a MLP with 1024*/*512*/*256 ReLU units per layer respectively, while the output is a sigmoidal unit as we want binary classification. The 1D CNN architecture is shown in Fig. 2, while the 2D architecture is depicted in Fig. 3.

**Fig. 4:**
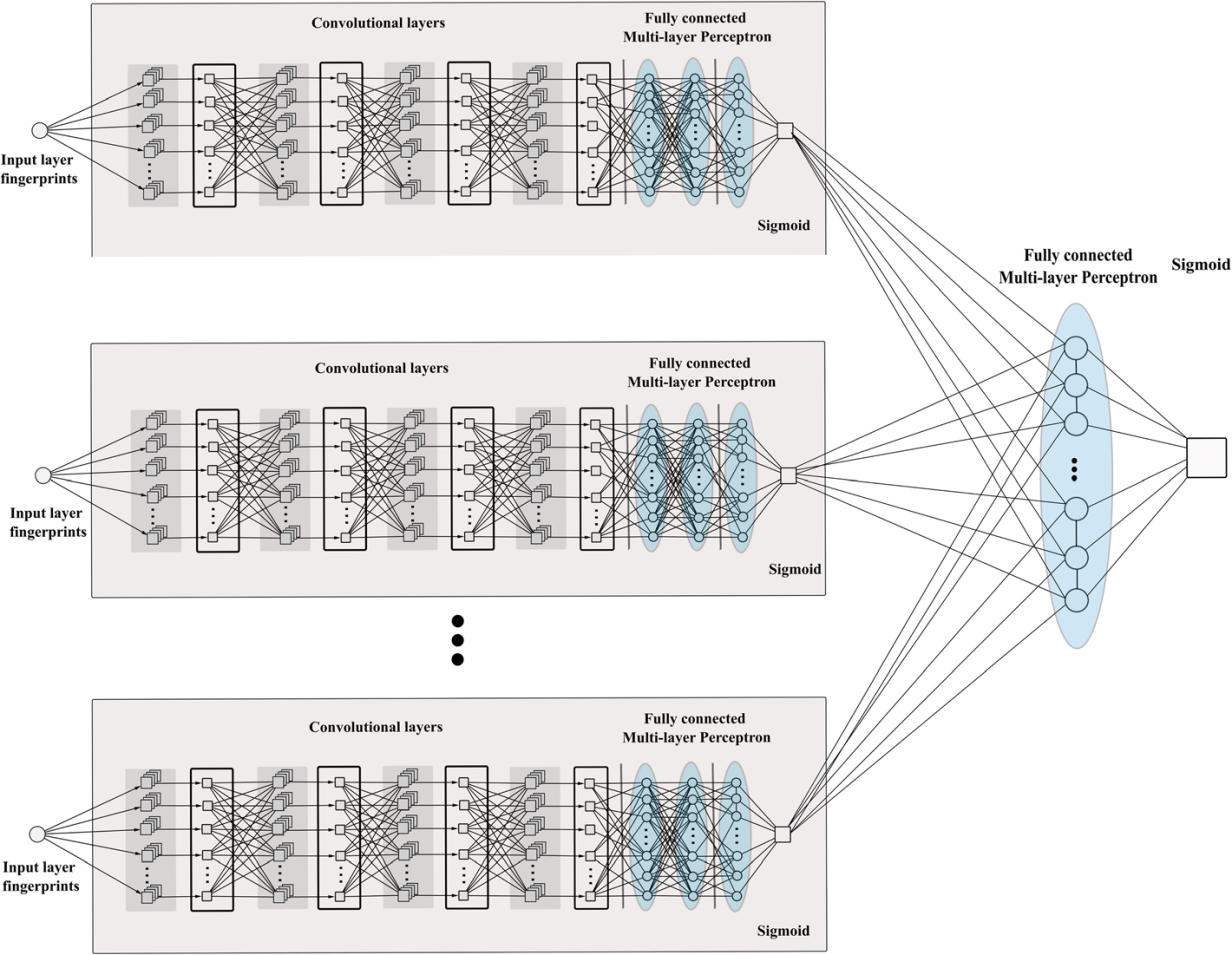
Tuned-MLP-Out. The complex architecture with MLP classifier

The last two architectures presented in this work are ensemble classifiers using the outputs of the 1D CNNs. 2D CNNs are characterized in general by a very low loss value, and provide a very high AUC value but their accuracy is never the highest one. Moreover, these networks exhibit a sort of inertia that is they attain both high sensitivity and balanced accuracy but it is not possible to stress their performance towards either mature or early screening task. This is mainly due to the intrinsic redundancy of the input fingerprints regardless the best performing combination. On the contrary, 1D CNN are more flexible architectures than the 2D ones, and are implemented by low capacity models. Such networks suffer from the use of a single fingerprint, which might not encode properly the core bioactivity features of the compound due to its particular generation algorithm. As a consequence we resorted to two kinds of ensemble classifiers: the first one, which has been called *Voting*, is a pure voting mechanism where the output labels from the best performing 1D CNN for each fingerprint type are collected, and the final label is the one provided by the majority of the voting classifiers. The second scheme (called *Tuned-MLP-Out*) is a more refined version of the pure voting mechanism, where we trained again from scratch seven 1D CNNs, one for each fingerprint type, with 512*/*256*/*128*/*64 filters per layer, and the same MLP arrangement as regards their classification stage. All of them are connected in parallel as inputs of a unique MLP layer through the probability values associated to the sigmoidal outputs. Just three ReLU units were needed for the final classification layer. The whole *Tuned-MLP-Out* architecture is reported in Fig. 4.

### Hyperparameter tuning

Hyperparameters tuning was performed as a grid search in the following sets of values. Convolutional filters tested were [1024*,*512*,*256*,*128*,*64*,*32*,*16] in combination with all Keras padding value; learning rate were multiplied by 10 in the ranges [10^−6^*,* 1;2 · 10^−5^*,* 0*.*2]. Dropout probabilities where in the range [0*.*2*,*0*.*9] with step 0*.*1, all the available optimizers in Keras were tested. Bi-dimensional tested kernel sizes were {(20*,*2)*,* (20*,*1)*,* (15*,*2)*,* (15*,*1)*,* (5*,*2)*,* (5*,*1)*,* (4*,*2)*,* (4*,*1)*,* (3*,*2)*,* (3*,*1)}, while 1D tested kernels were {2*,*3*,*4}. Batch sizes were doubled in the range [8*,*128]. Early sopping was used to devise training epochs, and model checkpoint to save the best model after each epoch. Hyperparameter optimization took about 100 hours to be accomplished on a GPU NVIDIA TITAN Xp 12 GB, 3840 CUDA Cores, while each Tuned-MLP-Out experiment took about 2 hours.

## Data Availability

The datasets analysed during the current study are available in the ChEMBL repository, [https://www.ebi.ac.uk/chembl/target report card/CHEMBL308/, https://www.ebi.ac.uk/chembl/target report card/CHEMBL1907602/, https://www.ebi.ac.uk/chembl/target report card/CHEMBL2815/, https://www.ebi.ac.uk/chembl/target report card/CHEMBL5464/, https://www.ebi.ac.uk/chembl/target report card/CHEMBL4282/, https://www.ebi.ac.uk/chembl/target report card/CHEMBL5932/] The curated data obtained after preprocessing are available from the corresponding author on reasonable request.

## Abbreviations

VS: Virtual screening
ADMET: Absorption, distribution, metabolism, excretion and toxicity
ML: Machine learning
SVM: Support vector machine
RF: Random forest
CNN: Convolutional neural network
DNN: Deep neural network
SMILES: Simplified molecular input lineentry system
CDK1: Cyclin-dependent kinase 1
QSAR: Quantitative structure-activity relationship
MLP: Multi layer perceptron
*b*ACC: Balanced accuracy
TP: True positive
P: Positive
TN: True negative
N: Negative
MCC: Matthews correlation coefficient
AUC: Area under the curve
EF: Enrichment factor
IC50: Half maximal inhibitory concentration
WCSS: Within cluster sum of squares
ReLU: Rectified linear unit
